# The Common Structural Architecture of *Shigella flexneri* and *Salmonella typhimurium* Type Three Secretion Needles

**DOI:** 10.1371/journal.ppat.1003245

**Published:** 2013-03-21

**Authors:** Jean-Philippe Demers, Nikolaos G. Sgourakis, Rashmi Gupta, Antoine Loquet, Karin Giller, Dietmar Riedel, Britta Laube, Michael Kolbe, David Baker, Stefan Becker, Adam Lange

**Affiliations:** 1 Department of NMR-based Structural Biology, Max Planck Institute for Biophysical Chemistry, Göttingen, Germany; 2 Laboratory of Chemical Physics, National Institute of Diabetes and Digestive and Kidney Diseases, National Institutes of Health, Bethesda, Maryland, United States of America; 3 Department for Cellular Microbiology, Max Planck Institute for Infection Biology, Berlin, Germany; 4 Laboratory for Electron Microscopy, Max Planck Institute for Biophysical Chemistry, Göttingen, Germany; 5 Core Facility Microscopy, Max Planck Institute for Infection Biology, Berlin, Germany; 6 Department of Biochemistry, University of Washington, Seattle, Washington, United States of America; Osaka University, Japan

## Abstract

The Type Three Secretion System (T3SS), or injectisome, is a macromolecular infection machinery present in many pathogenic Gram-negative bacteria. It consists of a basal body, anchored in both bacterial membranes, and a hollow needle through which effector proteins are delivered into the target host cell. Two different architectures of the T3SS needle have been previously proposed. First, an atomic model of the *Salmonella typhimurium* needle was generated from solid-state NMR data. The needle subunit protein, PrgI, comprises a rigid-extended N-terminal segment and a helix-loop-helix motif with the N-terminus located on the outside face of the needle. Second, a model of the *Shigella flexneri* needle was generated from a high-resolution 7.7-Å cryo-electron microscopy density map. The subunit protein, MxiH, contains an N-terminal α-helix, a loop, another α-helix, a 14-residue-long β-hairpin (Q51–Q64) and a C-terminal α-helix, with the N-terminus facing inward to the lumen of the needle. In the current study, we carried out solid-state NMR measurements of wild-type *Shigella flexneri* needles polymerized *in vitro* and identified the following secondary structure elements for MxiH: a rigid-extended N-terminal segment (S2-T11), an α-helix (L12-A38), a loop (E39-P44) and a C-terminal α-helix (Q45-R83). Using immunogold labeling *in vitro* and *in vivo* on functional needles, we located the N-terminus of MxiH subunits on the exterior of the assembly, consistent with evolutionary sequence conservation patterns and mutagenesis data. We generated a homology model of *Shigella flexneri* needles compatible with both experimental data: the MxiH solid-state NMR chemical shifts and the state-of-the-art cryoEM density map. These results corroborate the solid-state NMR structure previously solved for *Salmonella typhimurium* PrgI needles and establish that *Shigella flexneri* and *Salmonella typhimurium* subunit proteins adopt a conserved structure and orientation in their assembled state. Our study reveals a common structural architecture of T3SS needles, essential to understand T3SS-mediated infection and develop treatments.

## Author Summary

Gram-negative bacteria use a molecular machinery called the Type Three Secretion System (T3SS) to deliver toxic proteins to the host cell. Our research group has recently solved the structure of the extracellular T3SS needle of *Salmonella typhimurium*. Employing solid-state NMR, we could determine local structure parameters such as dihedral angles and inter-nuclear proximities for this supramolecular assembly. Concurrently, a high-resolution cryo-electron microscopy density map of the T3SS needle of *Shigella flexneri* was obtained by Fujii *et al.* Modeling of the *Shigella* needle subunit protein to fit the EM density produced a model incompatible with the atomic model of the *Salmonella* needle in terms of secondary structure and subunit orientation. Here, we determined directly the secondary structure of the *Shigella* needle subunit using solid-state NMR, and its orientation using *in vitro* and *in vivo* immunogold labeling in functional needles. We found that *Shigella* subunits adopt the same secondary structure and orientation as in the atomic model of *Salmonella*, and we generated a homology model of the *Shigella* needle consistent with the EM density. Knowing the common T3SS needle architecture is essential for understanding the secretion mechanism and interactions of the needle with other components of the T3SS, and to develop therapeutics.

## Introduction

The Type Three Secretion System (T3SS) is a secretion machinery used by Gram-negative bacteria to deliver virulence factors into eukaryotic host cells [1]–[2]. T3SS are found in animal and plant pathogens [3]. Human pathologies involving type-three secretion include bacillary dysentery (*Shigella*), intestinal inflammations and diarrhea (*Salmonella*, *Escherichia, Aeromonas*), melioidosis (*Burkholderia*), the plague (*Yersinia*), cholera (*Vibrio*) and pneumonia (*Pseudomonas*) [4]. Shigellosis, the pathology associated with *Shigella* infections, is responsible for 165 million infections and 1.5 million deaths annually [5]–[7].

T3SS are formed by the assembly of ∼20–25 different proteins, some of these in multiple copies. They consist of a hollow needle and a basal body which anchors the needle to both bacterial membranes [2], [8]–[10]. The needle extends into the extracellular space and ends in a tip that may make contact with the host cell [11]–[14]. Upon contact, translocator proteins form a pore through which effector proteins enter and subsequently alter host cell processes during infection [7]–[8]. The needle is assembled from multiple copies of a subunit protein, namely MxiH in *Shigella*, PrgI in *Salmonella* and BsaL in *Burkholderia* bacteria. Earlier studies of *Shigella flexneri* T3SS needles by X-ray fiber diffraction and cryo-electron microscopy (cryoEM) showed that the MxiH subunits are disposed in a helical arrangement, with 5.6 subunits per turn and a 24-Å helical pitch [15]. The atomic structures of C-terminal truncation mutants, resulting in non-functional soluble needle subunits, were determined for BsaL^CΔ5^ by solution-state NMR [16], MxiH^CΔ5^ by X-ray crystallography [17], and PrgI^CΔ5^ by solution-state NMR [18]. All three structures exhibit two α-helical segments separated by a loop at the P-(S/D)-(D/N)-P motif.

**Figure 1:**
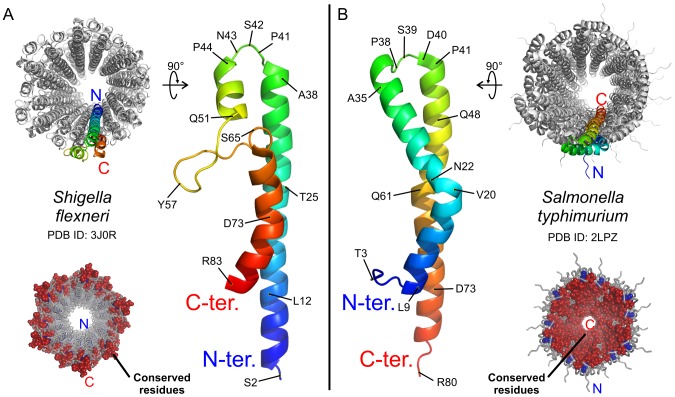
Proposed topologies for T3SS needle proteins in the needle assembly. *A*, Model of *Shigella flexneri* T3SS needle based on a 7.7-Å cryoEM map, according to Ref. [19], PDB ID: 3J0R. The structure of the MxiH subunit protein contains an α-helix (S2-A38), a loop (K39-P44), an α-helix (Q45-Y50), a 14-residue-long β-hairpin (Q51–Q64) and an α-helix (S65-R83) with a kink at D73. The N-terminus faces inward to the lumen of the needle. *B*, Solid-state NMR atomic model of the *Salmonella typhimurium* T3SS needle according to Ref. [20], PDB ID: 2LPZ. The structure of the PrgI subunit protein comprises a rigid-extended conformation (T3-Y8), an α-helix (L9-A35) with a kink at N22, a loop (A36-P41), and an α-helix (A42-R80). The N-terminus is located at the surface of the needle. Top views of the needle assemblies are shown in the top corners. The structures in the bottom corners present the location of highly conserved residues that were identified from multiple sequence alignment (Fig. 2). The atoms of conserved amino acids are represented as spheres and are colored respectively blue or red depending if their amino acid belongs to the N- or the C-terminal region. Conserved residues are lining the lumen of the needle in the *Salmonella* model but are exposed to the extracellular milieu in the *Shigella* model.

In order to gain further insight into the function of Type Three Secretion Systems, it is necessary to obtain an atomic structure of wild-type (WT) needles in their assembled state. The pseudoatomic T3SS needle architecture of *Shigella* was modeled by Deane and coworkers [17] by docking semi-rigid MxiH^CΔ5^ crystal monomers into a 16-Å cryoEM density map [15], with a topology where the N-terminus of the MxiH subunit is facing the inside of the tubular structure. More recently, a refined model of the *Shigella* MxiH needle was presented by Fujii and coworkers [19] based on a 7.7-Å cryo-electron microscopy (cryoEM) map. In this model (Fig. 1*A*), the segment ranging from Q51 to Q64, observed as α-helical in the X-ray structure [17], was remodeled as a β-hairpin structure during the molecular docking, while preserving the inwards-facing N-terminus. We have recently presented an atomic model of the *Salmonella typhimurium* T3SS needle [20] based on solid-state NMR distance restraints collected directly on assembled needles and symmetry from scanning transmission electron microscopy (STEM) data [21]. In this atomic model (Fig. 1*B*), the N-terminus of the subunit is located at the surface of the needle, as confirmed by immunogold labeling experiments performed both *in vitro* and *in vivo* [20].

**Figure 2:**
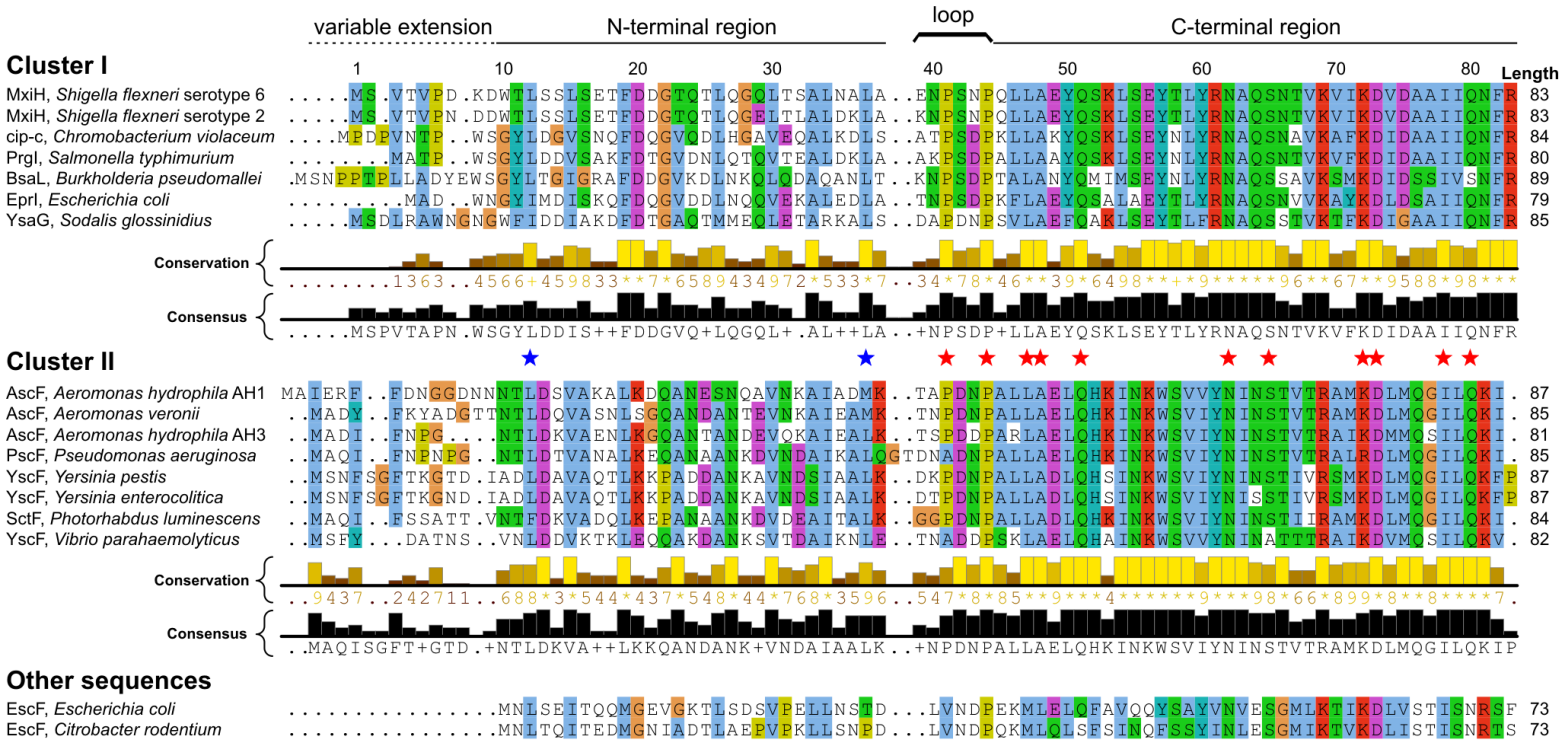
Multiple sequence alignment of T3SS needle proteins. Conserved residues are colored according to amino acid type following the Clustal X color scheme [75]. Highly conserved residues (≥12 identical amino acids in both clusters) are highlighted with a star (N-terminal region: blue; C-terminal region: red). The numbering of residues corresponds to the sequence of MxiH. Database accession identifiers for the primary sequences are given in supplementary Table S3 in Text S1.

Although the two proposed models show fundamental differences in their topology, MxiH and PrgI present a high sequence identity (>60%, Fig. 2). From these two models, strong experimental predictions regarding the orientation of termini and the secondary structure present in *S. flexneri* T3SS needles can be made and experimentally verified. In the current study, we thus present solid-state NMR data and immunogold labeling results obtained on WT needles of *S. flexneri* which allow us to address the discrepancy between the two models and establish the common architecture of T3SS needles.

## Results

### *Shigella flexneri* T3SS needle MxiH subunits are rigid and highly ordered in the assembled state

**Figure 3:**
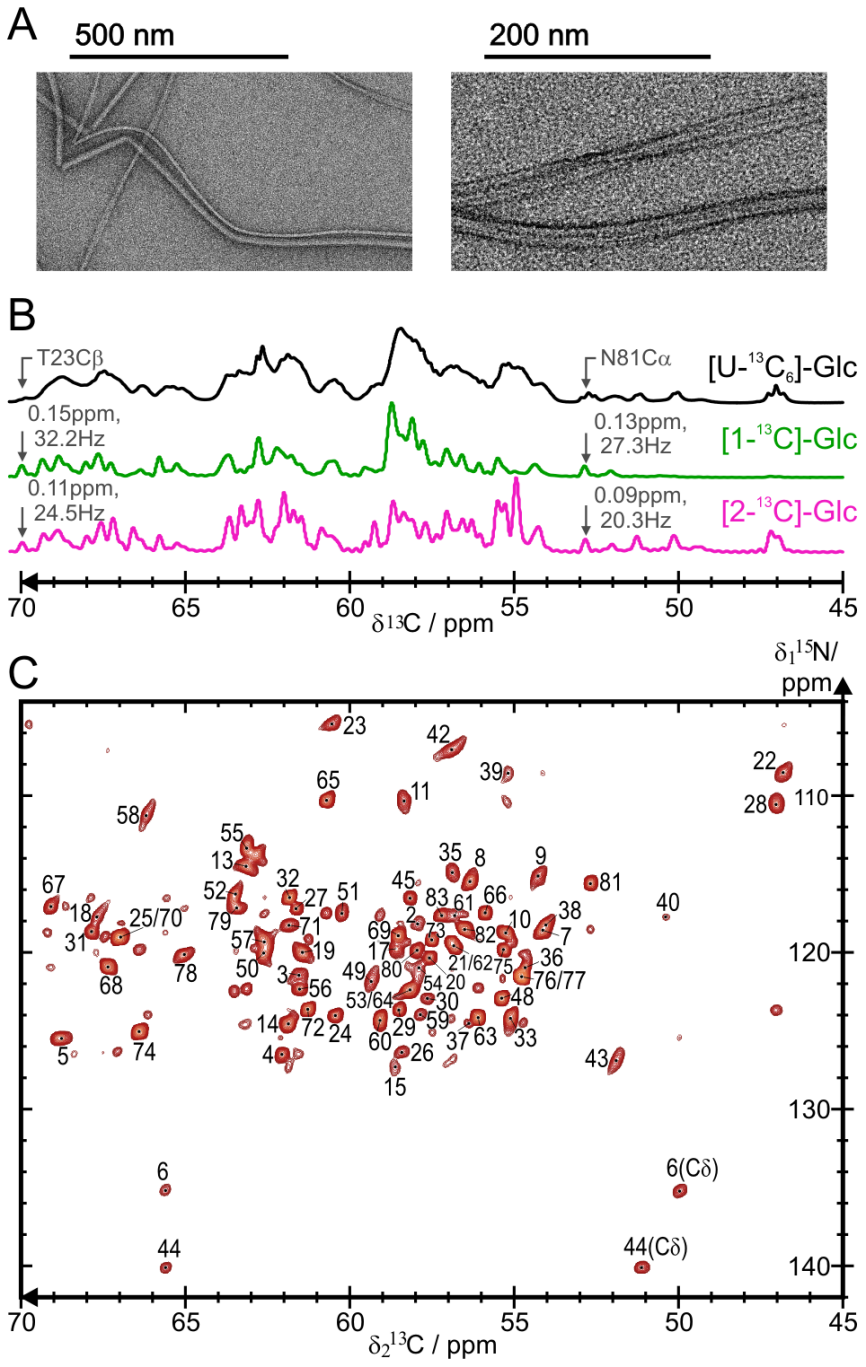
Solid-state NMR spectroscopy of *Shigella flexneri* MxiH needles. *A*, Transmission electron microscopy images of WT *Shigella flexneri* needles used for solid-state NMR measurements. *B*, 1D ^13^C cross-polarization spectra of [uniform-^13^C_6_]glucose (Glc)-labeled (black), [1-^13^C]Glc-labeled (green) and [2-^13^C]Glc-labeled (magenta) needles. Representative line-widths are indicated for two resonances (T23Cβ and N81Cα) for the sparsely-labeled samples. The free-induction decay of the signal was recorded for a total of 8192 scans, with signal remaining after an acquisition time of 60 ms. No apodization function was employed in the processing. *C*, N-Cα spectrum of [2-^13^C]Glc-labeled needles. The 2D spectrum correlates backbone amide nitrogen frequencies (δ_1_^15^N) to backbone Cα frequencies (δ_2_^13^C). N-Cα cross-peaks are numbered according to the MxiH amino acid sequence and N-Cδ cross-peaks of prolines are indicated. Unmarked cross-peaks correspond to sequential correlations. Spectra were recorded at a magnetic field of 21.1 T (850 MHz ^1^H resonance frequency) at 5.5°C.

The protocol developed for the *in vitro* preparation of functional *Salmonella* T3SS needles [20] was employed to produce WT *Shigella* T3SS needles (Fig. 3*A*). The needles were analyzed by magic-angle spinning solid-state NMR. Solid-state NMR is a method of choice [22]–[24] to obtain atomic-level structural information on insoluble proteins [25]–[28], including supramolecular assemblies [29]–[33], and can be employed directly on MxiH needles. The measurement of ^13^C cross-polarization spectra (Fig. *3B*) reveals that the recombinant needle samples are structurally highly homogeneous. The ^13^C line-widths measured for [1-^13^C]glucose (Glc)- and [2-^13^C]Glc-labeled needles range from 0.09 to 0.25 ppm (between 20 and 55 Hz on an 850 MHz spectrometer), on par with other protein samples showing the highest resolution in solid-state NMR: microcrystalline GB1 [34]–[35], the HET-s(218–289) prion domain [36], and *S. typhimurium* PrgI T3SS needles [20], [37].

The solid-state NMR assignment of ^13^C and ^15^N chemical shifts (supplementary Table S2 in Text S1) was carried out using standard experiments, 2D ^13^C-^13^C, ^15^N-^13^C and ^15^N-(^13^C)-^13^C experiments. Due to the high spectral resolution of the sample, most of the assignment could be carried out on uniformly-labeled ([U-^13^C_6_]Glc) needles using 2D spectroscopy. An example of the spectral resolution obtained in [U-^13^C_6_]Glc needles is presented in a ^13^C-^13^C proton-driven spin diffusion (PDSD) spectrum (supplementary Fig. S1 in Text S1). A near-complete resonance assignment (96%) was obtained using an extensive dataset of 2D spectra (supplementary Table S1 in Text S1) recorded on [U-^13^C_6_]Glc and ^13^C spin diluted (produced with [1-^13^C]Glc- and [2-^13^C]Glc) T3SS needles. In sparsely-labeled samples, the dilution of ^13^C spins results in an improved ^13^C resolution due to the removal of one-bond ^13^C-^13^C dipolar and *J*_CC_ couplings, and in the simplification of spectra [37] (Fig. 3*C*). For instance, the complementarity of the [1-^13^C]Glc- and [2-^13^C]Glc labeling patterns [37]–[40] enabled the facile assignment of the repetitive LSSLS motif (L12-S16) of MxiH.

Residues extending from S2 to R83 in the MxiH sequence are all observed in cross-polarization based solid-state NMR spectra, indicating a high structural rigidity. Consequently, with the exception of Met1 detected in an INEPT ^1^H-^13^C spectrum, all residues in the MxiH subunit protein exhibit a rigid conformation.

No peak doubling could be observed in solid-state NMR spectra (Figs. 3*C* and S1 in Text S1), denoting the absence of polymorphism in the needle samples. This is in contrast to the X-ray structure of monomeric MxiH^CΔ5^ (PDB ID: 2CA5) where two distinct conformations of MxiH are present in the unit cell [17], molecules A and B. Conformation B, which was not consistent with the low-resolution EM density, had been attributed either to crystal packing or to a different activation state of the needle [17].

### *Shigella flexneri* and *Salmonella typhimurium* needle proteins adopt a similar conformation in the assembled state

**Figure 4:**
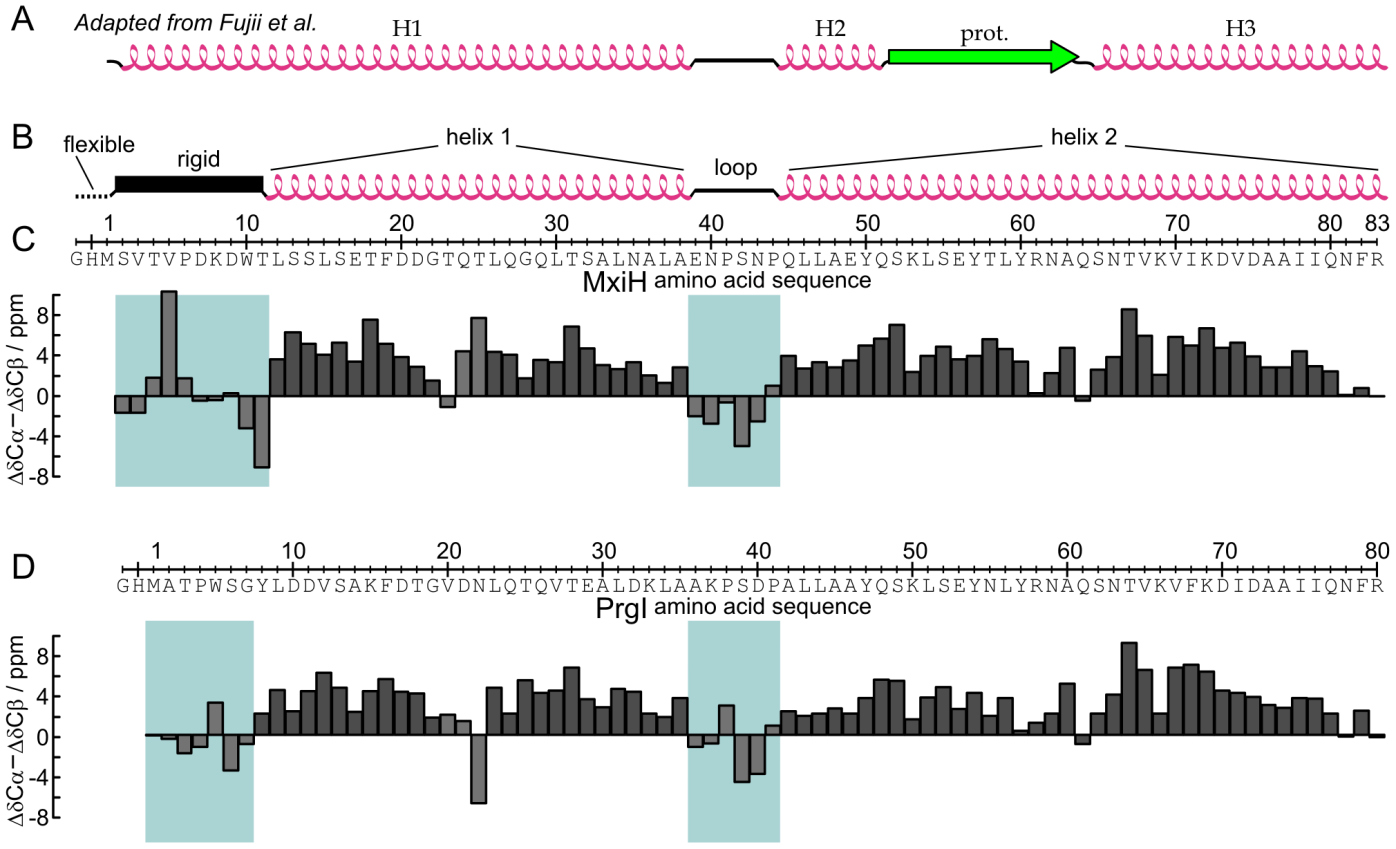
Identification of secondary structure elements. *A*, Secondary structure elements identified in the cryoEM model of Fujii *et al.*; adapted from Fig. 1e in Ref. [19] where H1, H2, and H3 indicate the position of α-helices and “prot” that of the β-hairpin between H2 and H3. *B*, Secondary structure elements of MxiH needles determined by solid-state NMR (this study). *C*, Secondary chemical shifts (ΔδCα – ΔδCβ) of the *Shigella flexneri* MxiH protein in the assembled needle. Regions which do not present α-helical propensity are highlighted. *D,* Secondary chemical shifts of the *Salmonella tympimurium* PrgI protein in the assembled needle taken from Ref. [37].

As backbone chemical shifts are strongly correlated to secondary structure [41]–[43], the secondary structure elements present in the MxiH subunit can be identified from our assignment (Figs. 4*B and 4C*). Positive values of secondary chemical shifts (Δδ) are indicative of α-helical propensity while negative values indicate β-sheet propensity [43]. The analysis of Δδ thus reveals two stretches of predominantly positive values corresponding to two long α-helices. The first α-helix is 27-residues long (L12 to A38), the second α-helix comprises 39 residues and extends up to the C-terminus (Q45 to R83). The intervening region of negative values (E39 to P44) corresponds to a rigid loop and contains the conserved P-(S/D)-(D/N)-P turn structure previously identified in structures of monomeric [16]–[18], [44] and assembled [19]–[20] T3SS needle subunits. Kinks or bends in the α-helices can be inferred from the isolated negative values of Δδ at T23 and Q64. The N-terminal segment from S2 to T11 is not α-helical but adopts instead a more extended conformation, as identified from the presence of negative Δδ values.

Local protein conformations can be directly compared on the basis of the secondary chemical shifts, as they are not influenced by amino acid type. The similarity in secondary chemical shifts between the MxiH and PrgI needles is remarkable (Figs. 4*C and 4D*), suggesting that the local structure of these needle subunits is highly similar. The agreement is especially apparent in the two α-helical regions, and the slight bend in the C-terminal helix occurs at the same position in both subunits, Q64 for MxiH corresponding to Q61 for PrgI. Two minor differences are observed: the kink appears at T23 for MxiH rather than at T25 (which would correspond to N22 in PrgI); more importantly, the rigid-extended region spans a larger region in MxiH, which has three additional residues, and the deviations of Δδ from random coil values are more pronounced. On the other hand, the secondary structure elements proposed in the model of Fujii *et al.* are not compatible with the experimental chemical shifts of MxiH (Fig. 4*A*). In order to quantitatively compare the cryoEM model to our chemical shift values, we back-predicted secondary chemical shifts from the cryoEM model (Fig. S2*B* in Text S1) using SPARTA+ [45]. It was reported that the back-prediction of solid-state NMR ^13^C chemical shifts in SPARTA produces outliers, i.e. predictions deviating from experimental shifts by more than 3 ppm, in approximately 4% of cases [46]. The number of ^13^C chemical shift outliers for the cryoEM-based model of Fujii *et al.*, 13.1%, is significantly higher, showing that its secondary structure cannot be reconciled with our NMR data.

**Figure 5:**
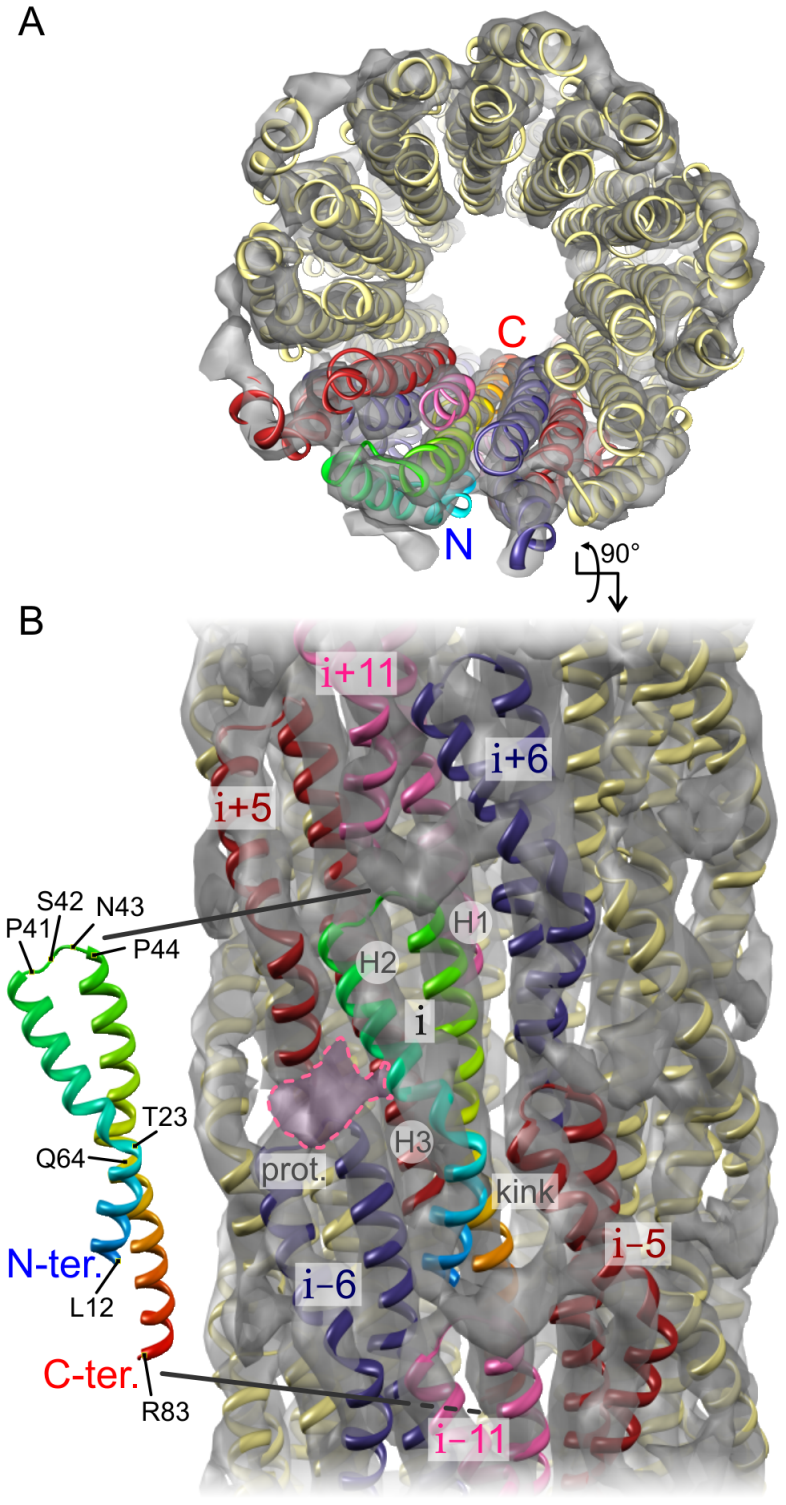
Structural model of the T3SS *Shigella* needle. *A*, Top and *B*, side views of the T3SS needle assembly of *Shigella flexneri*. The homology model of the 29-subunit MxiH needle assembly generated by Rosetta modeling (cartoon representation) shows a correlation of 0.66 with the 7.7-Å cryoEM density map (grey surface). Using the nomenclature of Fujii *et al.*, the EM density regions H1, H2 and H3 are indicated for the central subunit (i), as well as the kink in region H3. The first 11 N-terminal residues are not depicted due to the poor sequence homology of the rigid-extended segment (Fig. 2); however the protrusion in the EM map at subunit (i), labeled prot. and highlighted by a purple dashed contour trace, may be well explained by the N-terminus of subunit (i+5). The central subunit (i), colored as in Fig. 1*B*, forms a lateral interface with subunits (i±5), in red, and subunits (i±6), in dark blue. The axial interface is formed with subunits (i±11) shown in pink.

Despite the aforementioned discrepancies in secondary structure between the MxiH [19] and PrgI [20] models, there is a striking similarity in the shape of the 3D envelope of their constituting subunits (Fig. 1). We thus wanted to test whether the architecture identified in PrgI needles could explain all experimental data for MxiH needles, both solid-state NMR chemical shifts and the 7.7-Å cryoEM density map. We generated homology models of the MxiH needle by Rosetta modeling calculations [47] using the structure of the PrgI needle as a template. A representative model from this ensemble (Fig. 5) shows good overlap with the cryoEM density map (EM correlation of 0.66), demonstrating that the architecture found in the PrgI needle (Fig. 1*B*) is compatible with all experimental data. Using the nomenclature of Fujii *et al.*, the long C-terminal α-helix identified in PrgI occupies the density region H1 facing towards the needle pore in the cryoEM map of MxiH (Fig. 5*B*). The density regions H2 and H3 are filled by the N-terminal α-helix. The kink present in density region H3 corresponds to the kink identified at T23 from the analysis of secondary chemical shifts.

### The N-terminus of MxiH is exposed on the exterior of the assembly

**Figure 6:**
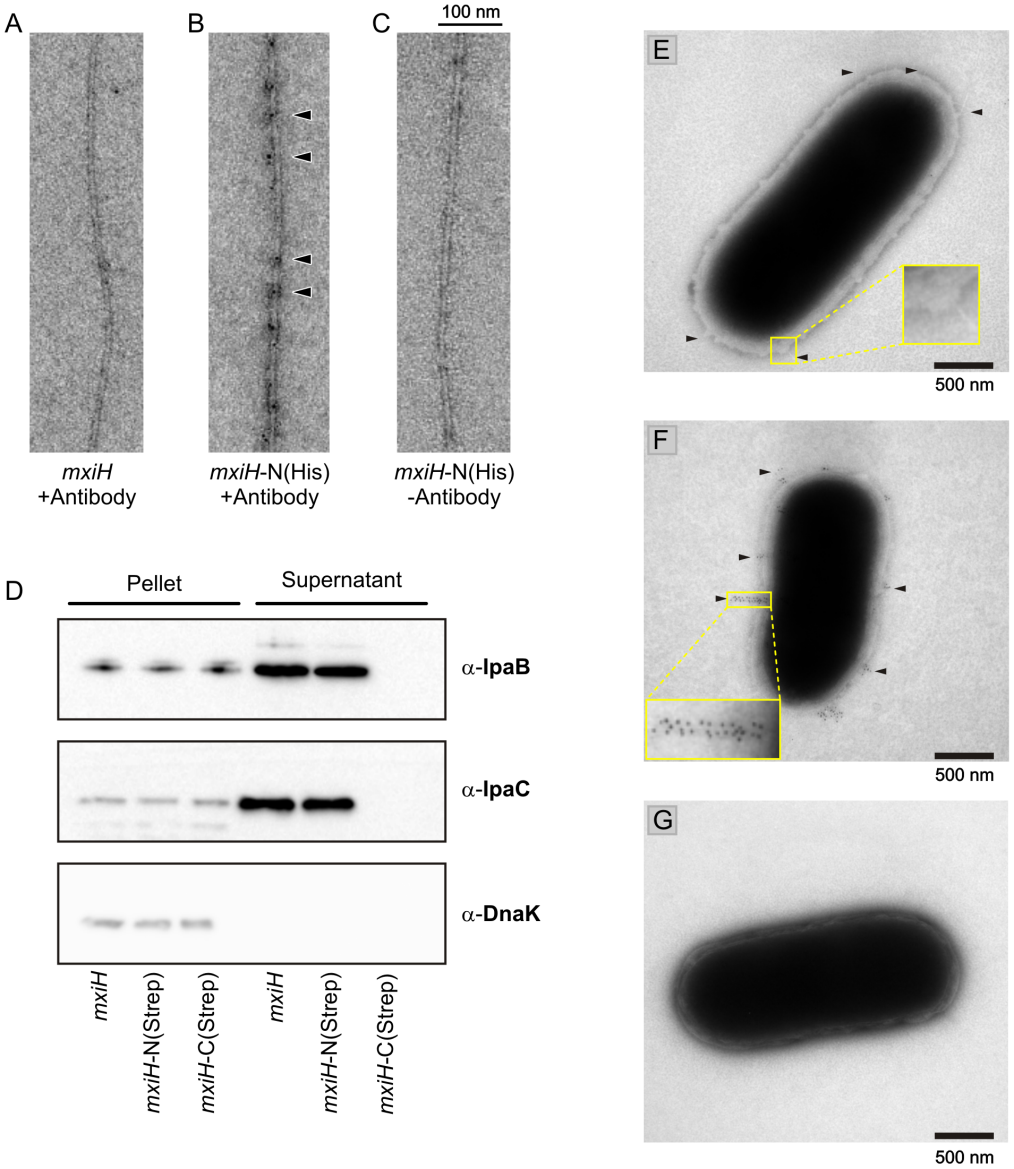
Localization of the MxiH subunit N-terminus *in vitro* and *in vivo*. *A*–*C*, Immunogold labeling of recombinant *Shigella flexneri* MxiH needles using a monoclonal Anti-His tag antibody. *A*, WT MxiH needles *B*, needles polymerized from N-terminal His tag fusion construct, *mxiH*-N(His), *C*, *mxiH*-N(His) needles labeled without primary Anti-His tag antibody. In *B*, some gold particles are indicated by arrows. Proteins expressed from a C-terminal His tag fusion construct did not polymerize. *D*, Effector protein secretion assay of *Shigella* strains used in Fig. *E*–*G* by addition of Congo Red (CR) followed by western blot analysis against IpaB, IpaC and DnaK. *E*–*G*, Non-polar *Shigella* mxiH-knockout cells (*ΔmxiH*) expressing *E*, wild-type *mxiH*, *F*, mxiH with N-terminal Strep-tag, *mxiH*-N(Strep) or *G*, MxiH with C-terminal Strep-tag, *mxiH*-C(Strep). In *E* and *F*, needles are indicated by arrows and two needles complexes are highlighted.

In order to determine the placement of the N- and the C-terminus in the MxiH needle, immunogold labeling experiments were performed both *in vitro* and *in vivo*. For *in vitro* experiments, the MxiH protein was expressed as an N-terminal His tag fusion construct, *mxiH*-N(His), and needles were polymerized. The tag is recognized by an Anti-His tag antibody which is imaged using protein A Gold (Fig. 6*B*). Immunogold labeling was not observed for WT needles lacking the His tag (Fig. 6*A*) and for *mxiH*-N(His) needles incubated without the Anti-His tag antibody (Fig. 6*C*), excluding the possibility that the labeling resulted from non-specific binding of the primary antibody or protein A Gold. *mxiH* expressed as a C-terminal His tag fusion construct prevented needle polymerization as previously reported [44]. Similar results were obtained for *in vivo* immunogold labeling experiments, where *ΔmxiH Shigella* cells were complemented with WT *mxiH* (Fig. 6*E*), an N-terminal Strep-tag fusion construct, *mxiH*-N(Strep), (Fig. 6*F*) or a C-terminal Strep-tag fusion construct, *mxiH*-C(Strep), (Fig. 6*G*). The strains expressing WT *mxiH* and *mxiH*-N(Strep) could assemble secretion-competent needles, as shown in a protein secretion assay (Fig. 6*D*), but the strain expressing *mxiH*-C(Strep) did not assemble any needle (Fig. 6*G*) and was not competent for secretion, consistent with previous mutagenesis data showing that C-terminal insertions compromise needle assembly and secretion function [48]. The strain expressing *mxiH*-N(Strep) formed needles decorated with gold particles. These results show that the MxiH subunit N-terminus is exposed at the surface of the needle and corroborate that MxiH needles adopt the topology found in the PrgI needle solid-state NMR structure [20] but not the topology of previous MxiH needle models [17], [19].

### The common structural architecture of *Shigella flexneri* and *Salmonella typhimurium* needles is reflected in sequence conservation patterns

As T3SS are employed by a variety of bacterial pathogens [3], we generated a multiple sequence alignment of T3SS needle proteins to evaluate the generality of the needle architecture. The sequences group in two clusters based on a pattern of highly conserved residues in the loop and C-terminal region (Fig. 2). Three distinct regions of the protein are identified according to the conservation level: the first ∼10 residues forming the N-terminal extension are poorly conserved (average conservation level of 2.1), the subsequent N-terminal region is moderately conserved (6.5), and the loop (7.2) and C-terminal region (8.7) are highly conserved. Both MxiH and PrgI belong to the same cluster, in contrast to the needle protein of *Yersinia pestis*, YscF, which shows lower sequence identity to MxiH (25%). Sequences for EscF proteins are more evolutionary distant and were not included in any of the clusters. They also lack the canonical P-(S/D)-(D/N)-P loop motif and the variable extension.

The loop and C-terminal region contain a large number of residues, 11, that are highly conserved in both clusters, compared to only two residues in the N-terminal region. Those highly conserved residues, indicated with stars in Fig. 2, are all located lining the lumen of the needle in the PrgI needle structure (Fig. 1*B*, bottom right corner); however, no conserved residues are present in the interior of the needle in the cryoEM MxiH model (Fig. 1*A*, bottom left corner).

## Discussion

In the current study, WT *S. flexneri* needle assemblies were prepared by *in vitro* polymerization and studied by solid-state NMR. The MxiH needle subunit is predominantly rigid (S2-R83). Our chemical shift assignment (supplementary Table S2 in Text S1) provides for the first time empirical information on the conformation of the MxiH termini in the context of the needle assembly, as the first 19 (molecule A) or 14 (molecule B) residues are disordered in the crystal structure of MxiH^CΔ5^, as well as the last 2 (molecule A) or 8 (molecule B) C-terminal residues [17].

The following secondary structure elements were identified for MxiH: a rigid-extended N-terminal conformation (S2-T11), followed by the N-terminal α-helix (L12-A38) with a kink at T23, a loop (E39-P44) and the C-terminal α-helix (Q45-R83). The conformation of *Shigella* MxiH and *Salmonella* PrgI subunits in their needle assembled state is highly similar, as revealed by the comparison of secondary chemical shifts (Figs. 4*C and 4D*). Equally, the structure of the PrgI subunit solved by solid-state NMR comprises a rigid-extended N-terminal conformation (T3-Y8), an α-helix (L9-A35) with a kink at N22, a loop (A36-P41), and a C-terminal α-helix (A42-R80).

In contrast, the chemical shifts observed in this work are incompatible with the secondary structure identified in the previous MxiH needle model [19] (Figs. 4*A* and S2 in Text S1). Two major discrepancies exist between this model (Fig. 1*A*) and the experimental chemical shifts: the first 11 N-terminal residues were previously modeled as an α-helix and the segment extending from residues Q51 to Q64 was previously modeled as a β-hairpin. One explanation for the discrepancy in secondary structure elements in MxiH subunits between the current study and the model of Fujii *et al.* is that the measurements were carried out on *S. flexneri* serotype 6 (this study) and serotype 2. Compared to serotype 6, the sequence of serotype 2 has the following mutations: D7N, K8D, Q29E, S32L, N35D, A36K and E39K. Since no amino acid substitutions between the two serotypes are located in the conjectured β-hairpin region of MxiH (Q51–Q64), rearrangement of the secondary structure of the C-terminal helix by these mutations would likely involve even greater alterations locally at the site of the mutation, which are not observed. The finding that PrgI sustains a significantly greater number of N-terminal substitutions while preserving a C-terminal conformation highly similar to MxiH further discredits this hypothesis. We conclude that the incompatibility between this model and the experimental data results from remodeling of the monomeric subunit structure to fit the cryoEM density map without the guidance of site-specific local structural information such as experimentally-determined dihedral angles and distance restraints.

As shown here and recently for the T3SS needle of *S. typhimurium* [20], solid-state NMR data can be used in complementation to cryoEM data for the generation or the validation of structural models of large assemblies. Owing to the high similarity of secondary chemical shifts between PrgI and MxiH, we generated a homology model of *S. flexneri* needles which accounts for all available experimental data, including the 7.7-Å cryoEM density map. Although the correlation to the cryoEM density is higher for the previous model by Fujii *et al.* (0.72) compared to our PrgI-based homology model (0.66), this is expected as no EM bias was used in the building of the current homology model. The previous model also has significantly higher full-atom energy after Rosetta refinement (Table 1). It is notable that the symmetry and helical parameters of the PrgI-based homology model are highly similar to the parameters derived from the cryoEM density map (Table 1), consistent with the view that *Shigella* and *Salmonella* needles are built upon a common architecture. From a methodological point of view, it is also interesting that both independent procedures, solid-state NMR in combination with results from STEM and Rosetta modeling — and high-resolution cryoEM, result in the same obtained symmetry. Furthermore, the fact that the homology model of PrgI fits well into the completely independently and simultaneously determined high-resolution cryoEM map (Fig. 5) supports the validity of the PrgI structure (Fig. 1*B*).

**Table 1:** Structural statistics of different needle models.

	Subunits per turn	Axial rise per monomer/Å	Needle Radius/Å	EM correlation	Full-atom energy*
This study	5.65	4.21	23.51	0.66	−184
Fujii *et al.* [19]	5.62	4.30	23.06	0.72	−133**

* Full-atom interface energy in Rosetta energy units, median value of the 10 lowest-energy models.

** After refinement of the PDB coordinates.

Using immunogold labeling, we confirmed that the N-terminus is oriented on the outside face of the needle assembly (Fig. 6). This subunit orientation is consistent with sequence conservation patterns observed by multiple sequence alignment (Fig. 2), where residues in the C-terminal helix are more frequently conserved, and with mutagenesis data, where sequence alterations are more easily tolerated at the N-terminus. Indeed, deletion of 2–5 residues or addition of 3–6 residues at the C-terminus results in dysfunctional needles as detected in effector secretion assays and Congo Red (CR) induction assays [19], [48] for MxiH, or in epithelial cell invasion assays [44] for PrgI. In contrast, deletion of 2–3 residues or addition of 3–7 residues at the N-terminus of MxiH subunits still produces functional needles (Ref. [19] and Fig. 6*D*). Moreover, PrgI subunits still assemble into functional T3SS needles when expressed with a deletion or addition of six residues at their N-terminus [44] as well as with a 8-residue N-terminal Strep-tag [20], as demonstrated by epithelial cell invasion assays. A deletion of eight N-terminal residues resulted in a *S. typhimurium* strain incapable of invasion [44], indicating that the two residues G7 and Y8 must be preserved to form the axial interface connecting subunits (i) and (i±11) and are necessary for the polymerization of PrgI subunits [20].

As the biological role of the T3SS needle is to ensure the secretion of effector proteins, it is consequent that the residues lining the lumen, directly involved in the transport of effectors, are highly conserved (Fig. 1*B*). On the other hand, the diversity of residues exposed at the surface of the needle can confer a selective advantage to pathogenic bacteria, which must evade recognition by host cells [7], [49]–[50]. Accordingly, the *mxiH* gene was shown to be the most variable gene in the virulence plasmid of *S. flexneri* [51].

In their assembled state, the only characteristic structural distinction observed between the homologous MxiH and PrgI proteins on the basis of the secondary structure analysis is the length of the N-terminal rigid-extended segment, which is composed of 10 residues for MxiH and six for PrgI. An important observation for the understanding of needle elongation is that T3SS needle proteins from different species are not interchangeable. For instance, Yop effector secretion could not be restored in a *yscF* null mutant strain of *Yersinia enterocolitica* by the expression of MxiH [52], which can be attributed to the low sequence identity of the two sequences (25%). However, despite the high sequence identity between MxiH and PrgI (>60%), a *Shigella flexneri mxiH* null mutant strain could not be complemented by PrgI and was not invasive due to the lack of needle formation [18]. Similarly, purified T3SS needles could be elongated *in vitro* from soluble monomer subunits of their own species, but not from subunits of other species, *Salmonella typhimurium* with MxiH* and *Shigella flexneri* with PrgI* [44]. Differences in the electrostatic surfaces from soluble monomeric subunit structures were suggested to explain the incompatibilities between needle subunits [18]. Our findings hint that the N-terminal rigid-extended segment could also play a crucial role in determining the species specificity. The excellent NMR spectral quality of needle samples produced by the *in vitro* preparation protocol [20] paves the way for the determination of high-resolution structures of MxiH filaments. Ongoing investigations in our group will thus address the question of the atomic structure of the rigid-extended N-terminal segment in MxiH needles.

In this study, it was found that *Shigella flexneri* and *Salmonella typhimurium* needles present a common architecture, providing a structural basis essential for the interpretation of previous functional data on type-three secretion in *Shigella*. Knowledge of the correct needle topology is essential to develop a key understanding of the operating mechanism of T3SS. The topology has an impact on the study of needle-tip interactions [53]–[57], needle-basal body interactions [58]–[62] and regulation of secretion [48], [63]–[66], which can contribute to the development of treatments against T3SS-mediated pathologies.

## Materials and Methods

### Sample preparation

MxiH was expressed and purified following the protocol established for *S. typhimurium* PrgI needles [20]. Similarly, the N-terminal hepta-Histidine (His) tag was cleaved using tobacco etch virus protease, releasing MxiH proteins containing the non-native N-terminal residues glycine and histidine. The protein concentration was raised to 0.2 mM during polymerization, which took place at 37°C for sixteen days. Approximately 20 mg of needle proteins were produced for each of three labeling schemes. All three samples were labeled with ^15^NH_4_Cl as nitrogen source and either D-[uniform-^13^C_6_]glucose ([U-^13^C_6_]Glc), D-[1-^13^C]glucose or D-[2-^13^C]glucose as carbon source.

### Solid-state NMR

MxiH needles were ultra-centrifuged and transferred into 4.0-mm magic-angle spinning rotors. Solid-state NMR experiments were conducted at 600 MHz, 800 MHz and 850 MHz ^1^H frequencies on Avance I and Avance III spectrometers (Bruker Biospin, Germany) at a spinning rate of 11 kHz and a temperature of 5.5°C. Additional experimental details can be found in supplementary Table S1 in Text S1. For the calculation of secondary chemical shifts (Δδ), average random coil chemical shifts were taken from Ref. [43].

### Immunogold labeling

Recombinantly-produced MxiH needles were labeled using an Anti-penta-Histidine tag monoclonal antibody (Invitrogen). The labeling was performed using a 1∶200 diluted antibody and 10-nm protein A Gold (Posthuma, Utrecht). Non-polar *Shigella mxiH*-knockout cells (*ΔmxiH*) were complemented with either wild-type *mxiH*, *mxiH* with N-terminal Strep-tag or *mxiH* with a C-terminal Strep-tag in pASK-IBA vectors (IBA lifesciences) and induced with 0.2 µg/ml anhydrotetracycline for 1 hr. The cells were fixed in 2% paraformaldehyde and immunogold labeled using 1∶40 diluted StrepMAB-Classic (IBA lifesciences) for 1 hr followed by 1∶40 diluted 12-nm gold-conjugated secondary antibody for 1 hr. Protein secretion assays were performed by addition of 20 µg/ml CR followed by western blot analysis against IpaB, IpaC and DnaK.

### Multiple sequence alignment

Sequences of commonly studied T3SS needle proteins were aligned using Clustal W version 2 [67] with default settings and the GONNET substitution matrix [68]. A first round of alignment revealed two clusters of highly conserved patterns in the loop and C-terminal region with the N-terminal regions presenting a higher variability. In a second round, the two sequence clusters were aligned as two separate groups. The conservation level and consensus sequence for each cluster were calculated using JalView [69]. Uniprot accession identifiers for the primary sequences are specified in supplementary Table S3 in Text S1. Pairwise sequence alignments used in the Rosetta modeling were generated using BLAST [70].

### Rosetta modeling

We used the Rosetta symmetric modeling framework [71] to generate a full 29-subunit model of MxiH based on the solid-state NMR structure of the PrgI needle (PDB ID: 2LPZ). This framework makes conformational sampling in symmetric systems more efficient by 1) only considering conformations that are consistent with the symmetry of the system and 2) performing a minimal number of energy and derivative evaluations by explicitly simulating only a subset of the interacting monomers and propagating conformational changes to symmetry-related subunits. The PrgI template was used to define the initial rigid-body placement and backbone conformation of the MxiH subunits as well as their relative orientation with respect to each other, therefore dictating the helical symmetry parameters. Unaligned residues at the N-termini of the subunits were remodeled using symmetric, Monte Carlo based 3- and 9-residue backbone fragment insertions in a coarse-grained representation of a helically-arrayed 29-subunit system. The entire structure, including all backbone, side chain and rigid body degrees of freedom was subsequently refined using a physically realistic all-atom energy function [72]. 1,000 homology-based models were generated of which the 10 lowest in energy showed strong convergence with 0.6 Å backbone RMSD for the entire complex, excluding the 11 residues comprised in the N-terminal variable extension. The final models did not diverge significantly from the starting PrgI template structure (0.8 Å backbone RMSD) and present almost identical helical parameters to the initial template (5.6 subunits/turn, 23.5 Å radius, 4.2 Å axial rise/monomer). Evaluation of the full-atom energy of the previous cryoEM model by Fujii *et al.* was done in the following manner: the full 29-subunit assembly was generated from the coordinates of the monomeric subunit using the symmetry-related operations described in the header of the PDB file (PDB ID: 3J0W), followed by the aforementioned symmetric refinement in Rosetta. The refinement led only to minor changes relative to the starting coordinates (<1 Å backbone RMSD for the rigid helical parts of the structure, <0.5 Å change in axial rise/monomer and radius, <10° in rotation/monomer). 100 models were generated in this manner and the median energy of the 10 lowest-energy models was reported (Table 1). Fitting of the homology model to the EM density map and calculation of the EM correlations were done using the program UCSF Chimera [73]–[74].

### BMRB

The ^15^N- and ^13^C-chemical shift assignments for MxiH, the T3SS needle protein of *Shigella flexneri*, were deposited at the Biological Magnetic Resonance Data Bank (BMRB) under accession number 18651.

## Supporting Information

Text S1 **Supporting information.** Figure S1, solid-state NMR ^13^C-^13^C spectrum of MxiH needles. Figure S2, back-prediction of secondary chemical shifts. Table S1, list of 2D solid-state NMR experiments. Table S2, chemical shifts of *Shigella flexneri* MxiH needles. Table S3, source of primary sequences for multiple sequence alignment.
(DOC)
