# Supplementary material for: The Common Structural Architecture of Shigella flexneri and Salmonella typhimurium Type Three Secretion Needles
Source: PLoS Pathog. 2013 Mar 21;9(3):e1003245. doi: 10.1371/journal.ppat.1003245 (PMC3605151; doi:10.1371/journal.ppat.1003245)
Supplement: Text S1 — Supporting information. Figure S1, solid-state NMR 13C-13C spectrum of MxiH needles. Figure S2, back-prediction of secondary chemical shifts. Table S1, list of 2D solid-state NMR experiments. Table S2, chemical shifts of Shigella flexneri MxiH needles. Table S3, source of primary sequences for multiple sequence alignment. (DOC) [file ppat.1003245.s001.doc]

**Figure S1.** Solid-state NMR ^13^C-^13^C spectrum of MxiH needles.

The aliphatic-carbonyl (left) and aliphatic-aliphatic (right) regions from the solid-state NMR ^13^C-^13^C correlation spectrum of [uniform-^13^C_6_]glucose-labeled MxiH needles are presented. The spectrum was recorded with a proton-driven spin diffusion (PDSD) mixing time of 50 ms on an 850 MHz spectrometer at 11 kHz MAS and at 5.5°C. Intra-residue cross-peaks are numbered according to the MxiH amino acid sequence, with the specific ^13^C atoms involved in the ^13^C-^13^C correlation indicated in subscript (F_1_, then F_2_). The C' symbol was omitted for correlations involving the backbone carbonyl. A few sequential correlations, especially in the C_α_-C_α_ region, were assigned but are not marked in the figure.


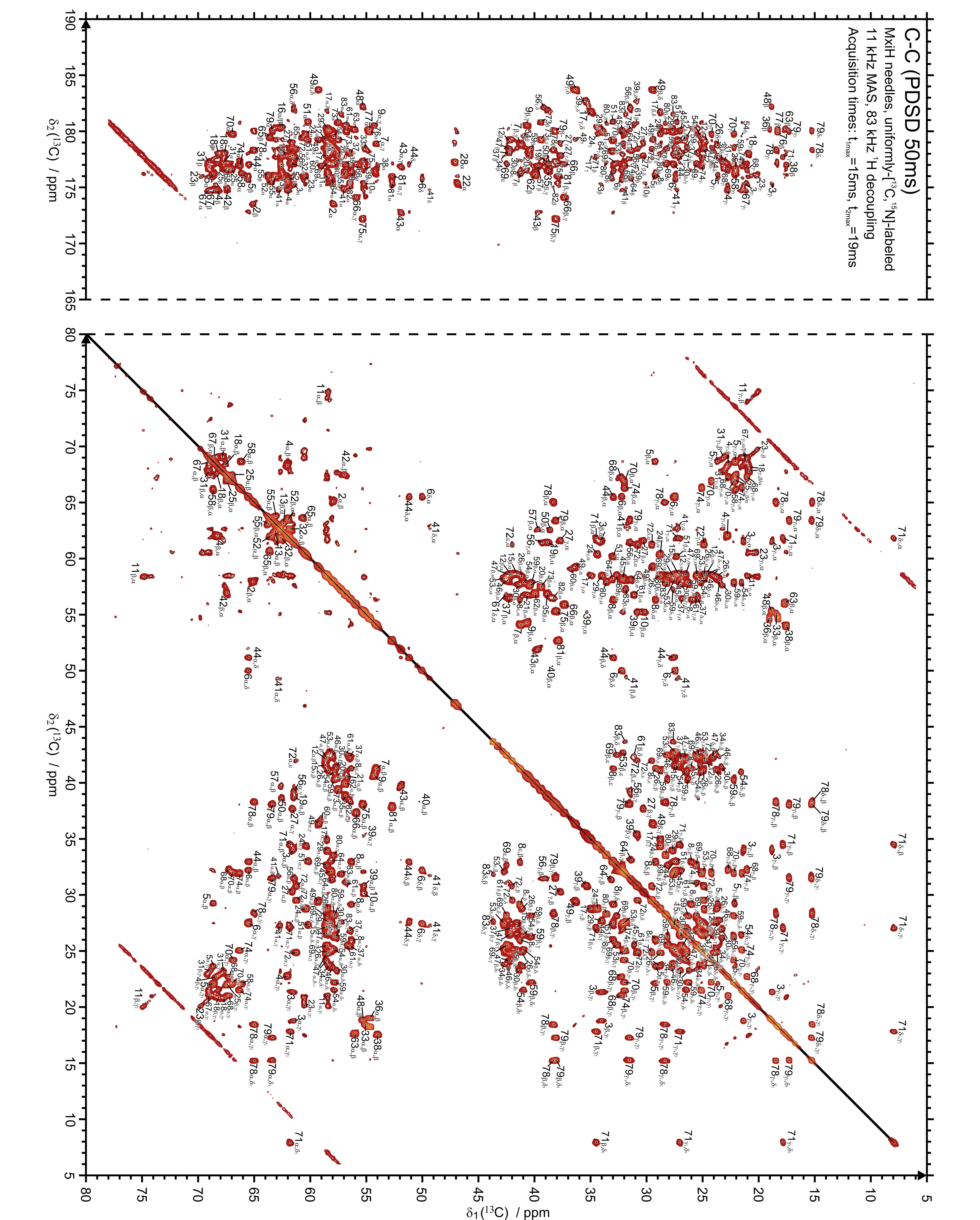


**Figure S2.** Back-prediction of secondary chemical shifts.

*A,* Secondary structure elements identified in the cryoEM model (adapted from of Fujii *et al.*, *Proc. Natl. Sci. Acad. U.S.A.*, **2012**, Fig. 1e) where H1, H2, and H3 indicate the position of α-helices and “prot” that of the β‑hairpin between H2 and H3. *B,* Secondary chemical shifts of the *Shigella flexneri* MxiH protein in the assembled needle measured by solid-state NMR (grey bars). Secondary chemical shifts back-predicted from the cryoEM model of Fujii *et al.* (blue squares) using SPARTA+. Error bars correspond to the estimated prediction error (s.d.).


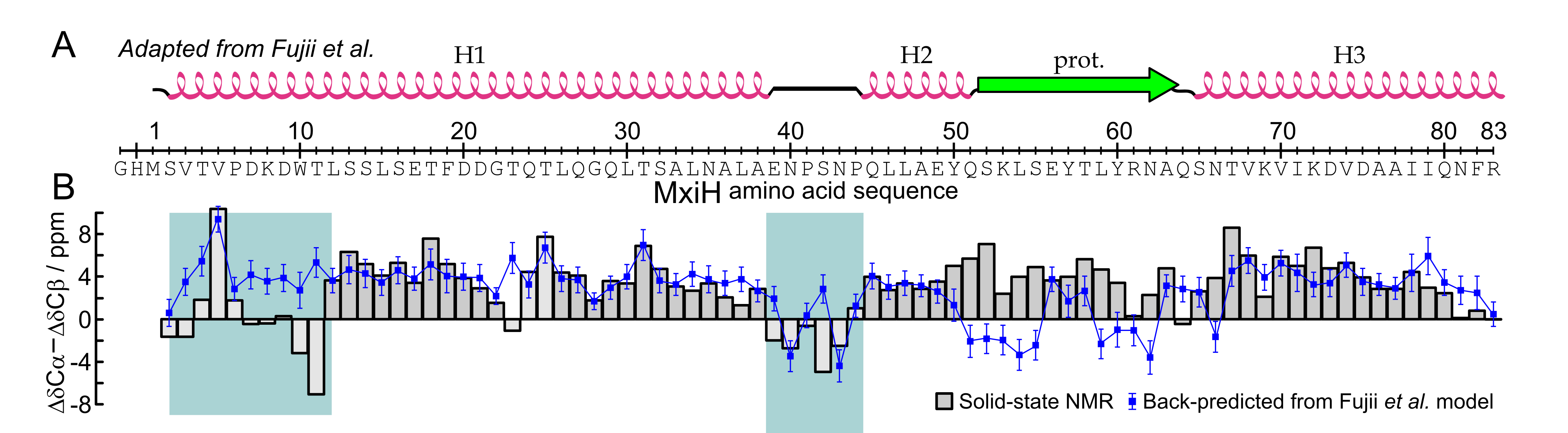


**Table S1.** List of 2D solid-state NMR experiments.

A ramped cross-polarization with contact time of 0.7-1.2 ms was used for the initial ^1^H-^13^C and ^1^H-^15^N transfers. For ^13^C-^13^C correlation experiments, carbon-carbon mixing was accomplished via proton-driven spin diffusion (PDSD) [1] with the indicated mixing times. Additionally, double quantum-single quantum correlation spectra were measured using the SPC5 sequence[2]. For ^15^N-^13^C correlation experiments, nitrogen-carbon transfer was carried out through n=1 double-quantum SPECIFIC-CP[3] with r.f. fields of 6.5 kHz on ^13^C and 4.5 kHz on ^15^N using a tangential ramp during 8.5 ms and 83.3 kHz of *cw* decoupling on ^1^H. Transfers were carried out in a band-selective fashion except for the N-CX experiment where broadband Hartman-Hahn matching was employed. In N-CA-CX and N-CO-CX experiments, a second transfer step to further carbon nuclei was carried out using DARR[4] for 70 ms. Proton decoupling with an r.f. frequency of 83.3 kHz was employed during evolution periods and acquisition, using either SPINAL-64[5] or SWf-TPPM[6] with a $R\bar{R}\bar{R}R$ supercycle[7] and a tangential sweep[8] (N = 11, sweep window d = 0.25, cut-off angle tco = 55°, phase angle θ = 15°). Carbon-nitrogen scalar couplings were removed by applying c.a. 2 kHz of waltz-16[9] decoupling on ^15^N during acquisition and ^13^C evolution periods, or with a 180° pulse on ^13^C during ^15^N evolution. The sample temperature was maintained at 5.5°C as determined from the ^1^H chemical shift of water in reference to DSS[10]. Maximal acquisition times in the indirect (t_1_) and direct (t_2_) dimensions are indicated as well as the total measurement time for each experiment.

|  |  |  |  |  |  |  | **Spectrometer frequency** |  | **Acquisition time** | | |  | **Total time** |  |
| --- | --- | --- | --- | --- | --- | --- | --- | --- | --- | --- | --- | --- | --- | --- |
|  | **Correlation** |  | **Mixing element** |  | **Labeling** |  | **ν_1_^1H^** |  | **t_1_** |  | **t_2_** |  |  |
|  |  |  |  |  |  |  | MHz |  | *m*s |  | *m*s |  |  |  |
|  | C-C |  | PDSD 50 ms |  | uniform |  | 850 |  | 15 |  | 19 |  | 1d 17h |  |
|  | N-CA |  | Selective CP |  | uniform |  | 850 |  | 15 |  | 15 |  | 24h |  |
|  | N-CA-CX |  | Selective CP, DARR 70 ms |  | uniform |  | 850 |  | 19 |  | 15 |  | 4d |  |
|  | N-CO |  | Selective CP |  | uniform |  | 850 |  | 19 |  | 15 |  | 12h |  |
|  | N-CO-CX |  | Selective CP, DARR 70 ms |  | uniform |  | 850 |  | 19 |  | 15 |  | 3d 14h |  |
|  | N-CX |  | Broadband H.-H. CP |  | uniform |  | 800 |  | 14 |  | 13.5 |  | 2d 9h |  |
|  | C-C |  | PDSD 75 ms |  | uniform |  | 800 |  | 12 |  | 20 |  | 2d |  |
|  | C-C |  | PDSD 200 ms |  | uniform |  | 800 |  | 14 |  | 14 |  | 3d |  |
|  | C-C DQ-SQ |  | SPC5 0.5 ms |  | uniform |  | 800 |  | 8 |  | 20 |  | 20h |  |
|  | H-C |  | INEPT |  | uniform |  | 800 |  | 12 |  | 25 |  | 1d 12h |  |
|  | C-C |  | PDSD 100 ms |  | uniform |  | 600 |  | 8 |  | 17 |  | 4d |  |
|  | C-C |  | PDSD 200 ms |  | uniform |  | 600 |  | 8 |  | 17 |  | 4d |  |
|  | N-CA |  | Selective CP |  | 1-glc |  | 800 |  | 14 |  | 17 |  | 1d 5h |  |
|  | C-C |  | PDSD 100 ms |  | 1-glc |  | 850 |  | 15 |  | 19 |  | 2d 10h |  |
|  | C-C |  | PDSD 400 ms |  | 1-glc |  | 850 |  | 17 |  | 21 |  | 4d 17h |  |
|  | C-C |  | PDSD 850 ms |  | 1-glc |  | 850 |  | 18 |  | 22 |  | 6d 16h |  |
|  | C-C |  | PDSD 700 ms |  | 1-glc |  | 800 |  | 8.5 |  | 14.5 |  | 7d 22h |  |
|  | N-CA |  | Selective CP |  | 2-glc |  | 850 |  | 17 |  | 30 |  | 15h |  |
|  | C-C |  | PDSD 50 ms |  | 2-glc |  | 850 |  | 15 |  | 21 |  | 1d 7h |  |
|  | C-C |  | PDSD 400 ms |  | 2-glc |  | 850 |  | 15 |  | 21 |  | 2d 23h |  |
|  | C-C |  | PDSD 850 ms |  | 2-glc |  | 850 |  | 16 |  | 21 |  | 6d 12h |  |
|  | C-C |  | PDSD 850 ms |  | 2-glc |  | 800 |  | 8.5 |  | 14 |  | 9d 1h |  |
|  | C-C |  | PDSD 300 ms |  | 2-glc |  | 600 |  | 17 |  | 21 |  | 4d 20h |  |
|  |  |  |  |  |  |  |  |  |  |  |  |  |  |  |

**References**

1. Szeverenyi NM, Sullivan MJ, Maciel GE (1982) Observation of spin exchange by two-dimensional fourier-transform ^13^C cross-polarization magic-angle spinning. Journal of Magnetic Resonance 47: 462-475.

2. Hohwy M, Rienstra CM, Jaroniec CP, Griffin RG (1999) Fivefold symmetric homonuclear dipolar recoupling in rotating solids: Application to double quantum spectroscopy. Journal of Chemical Physics 110: 7983-7992.

3. Baldus M, Petkova AT, Herzfeld J, Griffin RG (1998) Cross polarization in the tilted frame: assignment and spectral simplification in heteronuclear spin systems. Molecular Physics 95: 1197-1207.

4. Takegoshi K, Nakamura S, Terao T (2001) C-13-H-1 dipolar-assisted rotational resonance in magic-angle spinning NMR. Chemical Physics Letters 344: 631-637.

5. Fung BM, Khitrin AK, Ermolaev K (2000) An improved broadband decoupling sequence for liquid crystals and solids. Journal of Magnetic Resonance 142: 97-101.

6. Thakur RS, Kurur ND, Madhu PK (2006) Swept-frequency two-pulse phase modulation for heteronuclear dipolar decoupling in solid-state NMR. Chemical Physics Letters 426: 459-463.

7. Augustine C, Kurur ND (2011) Supercycled SWf-TPPM sequence for heteronuclear dipolar decoupling in solid-state nuclear magnetic resonance. Journal of Magnetic Resonance 209: 156-160.

8. Chandran CV, Madhu PK, Kurur ND, Brauniger T (2008) Swept-frequency two-pulse phase modulation (SWf-TPPM) sequences with linear sweep profile for heteronuclear decoupling in solid-state NMR. Magnetic Resonance in Chemistry 46: 943-947.

9. Shaka AJ, Keeler J, Frenkiel T, Freeman R (1983) An Improved Sequence for Broad-Band Decoupling - WALTZ-16. Journal of Magnetic Resonance 52: 335-338.

10. Bockmann A, Gardiennet C, Verel R, Hunkeler A, Loquet A, et al. (2009) Characterization of different water pools in solid-state NMR protein samples. Journal of Biomolecular NMR 45: 319-327.

**Table S2.** Chemical shifts of Shigella flexneri MxiH needles.

Chemical shifts are indicated in ppm relative to DSS.

|  | **N** | **C** | **C_α_** | **C_β_** | **C_γ_/C_γ1_** | **C_γ2_** | **C_δ_/C_δ1_** | **C_δ2_** | **C_ε_/C_ε1_** | **C_ε2_** | **C_ε3_** | **C_ζ_** | **C_ζ2_** | **C_ζ3_** | **N_δ2_/N_ε2_** | **N_ε_** | **N_ζ_** |
| --- | --- | --- | --- | --- | --- | --- | --- | --- | --- | --- | --- | --- | --- | --- | --- | --- | --- |
| **G-1** |  |  |  |  |  |  |  |  |  |  |  |  |  |  |  |  |  |
| **H0** |  |  |  |  |  |  |  | 120.0 | 136.6 |  |  |  |  |  |  |  |  |
| **M1** |  |  |  |  |  |  |  |  | 16.8 |  |  |  |  |  |  |  |  |
| **S2** | 118.0 | 173.5 | 57.9 | 65.1 |  |  |  |  |  |  |  |  |  |  |  |  |  |
| **V3** | 121.2 | 175.3 | 61.5 | 34.1 | 18.8 | 21.3 |  |  |  |  |  |  |  |  |  |  |  |
| **T4** | 126.3 | 174.7 | 62.1 | 68.4 |  | 22.7 |  |  |  |  |  |  |  |  |  |  |  |
| **V5** | 125.3 | 176.1 | 68.7 | 29.3 | 22.1 | 23.6 |  |  |  |  |  |  |  |  |  |  |  |
| **P6** | 135.1 | 175.7 | 65.6 | 32.2 | 27.4 |  | 50.0 |  |  |  |  |  |  |  |  |  |  |
| **D7** | 118.4 | 176.2 | 54.1 | 41.3 | 178.9 |  |  |  |  |  |  |  |  |  |  |  |  |
| **K8** | 115.3 | 176.4 | 56.4 | 33.0 | 26.0 |  | 29.2 |  | 41.3 |  |  |  |  |  |  |  | 33.0 |
| **D9** | 114.9 | 176.5 | 54.3 | 40.7 | 180.5 |  |  |  |  |  |  |  |  |  |  |  |  |
| **W10** | 118.5 | 175.4 | 55.3 | 30.1 | 109.7 |  | 128.9 | 130.8 |  | 139.0 | 121.2 |  | 113.2 | 120.7 |  |  |  |
| **T11** | 110.0 | 176.1 | 58.4 | 73.7 |  | 21.1 |  |  |  |  |  |  |  |  |  |  |  |
| **L12** | 120.0 | 179.2 | 58.6 | 42.1 | 27.0 |  | 26.5 | 24.6 |  |  |  |  |  |  |  |  |  |
| **S13** | 114.3 | 176.0 | 63.2 | 62.4 |  |  |  |  |  |  |  |  |  |  |  |  |  |
| **S14** | 124.4 | 177.8 | 61.8 | 62.2 |  |  |  |  |  |  |  |  |  |  |  |  |  |
| **L15** | 127.2 | 178.3 | 58.7 | 41.6 | 27.5 |  | 27.2 | 25.1 |  |  |  |  |  |  |  |  |  |
| **S16** | 113.4 | 180.3 | 62.5 | 62.7 |  |  |  |  |  |  |  |  |  |  |  |  |  |
| **E17** | 119.5 | 177.8 | 58.5 | 28.7 | 35.0 |  | 181.8 |  |  |  |  |  |  |  |  |  |  |
| **T18** | 117.6 | 178.0 | 67.7 | 68.3 |  | 20.8 |  |  |  |  |  |  |  |  |  |  |  |
| **F19** | 119.8 | 177.2 | 61.5 | 38.8 | 139.0 |  |  |  |  | 130.4 |  |  |  |  |  |  |  |
| **D20** | 120.1 | 178.0 | 57.6 | 40.4 | 178.3 |  |  |  |  |  |  |  |  |  |  |  |  |
| **D21** | 119.5 | 179.9 | 56.8 | 40.6 | 178.2 |  |  |  |  |  |  |  |  |  |  |  |  |
| **G22** | 108.2 | 175.4 | 46.8 |  |  |  |  |  |  |  |  |  |  |  |  |  |  |
| **T23** | 105.2 | 175.9 | 60.6 | 69.8 |  | 20.1 |  |  |  |  |  |  |  |  |  |  |  |
| **Q24** | 123.9 | 179.7 | 60.4 | 28.7 | 34.4 |  | 180.3 |  |  |  |  |  |  |  | 126.2 |  |  |
| **T25** | 118.8 | 176.6 | 67.0 | 67.5 |  | 22.0 |  |  |  |  |  |  |  |  |  |  |  |
| **L26** | 126.2 | 178.7 | 58.4 | 41.1 | 28.8 |  | 24.9 | 23.7 |  |  |  |  |  |  |  |  |  |
| **Q27** | 117.0 | 178.9 | 61.6 | 30.2 | 37.7 |  | 177.8 |  |  |  |  |  |  |  | 118.1 |  |  |
| **G28** | 110.4 | 177.3 | 47.1 |  |  |  |  |  |  |  |  |  |  |  |  |  |  |
| **Q29** | 123.5 | 180.2 | 58.5 | 27.7 | 33.9 |  | 180.0 |  |  |  |  |  |  |  | 112.0 |  |  |
| **L30** | 122.7 | 177.3 | 57.7 | 41.4 | 27.0 |  | 26.2 | 23.3 |  |  |  |  |  |  |  |  |  |
| **T31** | 118.5 | 177.1 | 67.8 | 69.1 |  | 23.1 |  |  |  |  |  |  |  |  |  |  |  |
| **S32** | 116.3 | 177.2 | 61.8 | 62.7 |  |  |  |  |  |  |  |  |  |  |  |  |  |
| **A33** | 124.0 | 178.4 | 55.1 | 18.4 |  |  |  |  |  |  |  |  |  |  |  |  |  |
| **L34** | 119.2 | 177.7 | 58.3 | 42.7 | 27.6 |  | 25.3 | 23.8 |  |  |  |  |  |  |  |  |  |
| **N35** | 114.6 | 177.1 | 56.9 | 38.8 | 176.5 |  |  |  |  |  |  |  |  |  | 115.8 |  |  |
| **A36** | 120.9 | 180.2 | 54.7 | 19.0 |  |  |  |  |  |  |  |  |  |  |  |  |  |
| **L37** | 124.3 | 178.0 | 56.5 | 42.2 | 26.9 |  | 25.4 | 25.5 |  |  |  |  |  |  |  |  |  |
| **A38** | 118.0 | 177.0 | 54.0 | 17.5 |  |  |  |  |  |  |  |  |  |  |  |  |  |
| **E39** | 108.3 | 176.8 | 55.2 | 30.9 | 35.4 |  | 182.8 |  |  |  |  |  |  |  |  |  |  |
| **N40** | 117.5 | 171.4 | 50.4 | 38.4 | 190.1 |  |  |  |  |  |  |  |  |  | 132.8 |  |  |

**Table S2 (continued).** Chemical shifts of Shigella flexneri MxiH needles.

|  | **N** | **C** | **C_α_** | **C_β_** | **C_γ_/C_γ1_** | **C_γ2_** | **C_δ_/C_δ1_** | **C_δ2_** | **C_ε_/C_ε1_** | **C_ε2_** | **C_ε3_** | **C_ζ_** | **C_ζ2_** | **C_ζ3_** | **N_δ2_/N_ε2_** | **N_ε_** | **N_ζ_** |
| --- | --- | --- | --- | --- | --- | --- | --- | --- | --- | --- | --- | --- | --- | --- | --- | --- | --- |
| **P41** | 130.0 | 174.8 | 62.8 | 31.8 | 27.2 |  | 49.3 |  |  |  |  |  |  |  |  |  |  |
| **S42** | 106.7 | 174.9 | 56.9 | 67.4 |  |  |  |  |  |  |  |  |  |  |  |  |  |
| **N43** | 126.8 | 172.7 | 51.9 | 39.7 | 176.9 |  |  |  |  |  |  |  |  |  | 111.3 |  |  |
| **P44** | 140.1 | 177.0 | 65.6 | 32.9 | 27.5 |  | 51.2 |  |  |  |  |  |  |  |  |  |  |
| **Q45** | 116.3 | 180.1 | 58.2 | 26.9 | 32.1 |  |  |  |  |  |  |  |  |  | 118.3 |  |  |
| **L46** | 121.3 | 178.8 | 58.0 | 42.3 | 27.7 |  | 25.2 | 23.5 |  |  |  |  |  |  |  |  |  |
| **L47** | 118.1 | 178.5 | 58.6 | 42.2 | 26.5 |  | 24.8 | 24.1 |  |  |  |  |  |  |  |  |  |
| **A48** | 122.7 | 182.2 | 55.3 | 18.8 |  |  |  |  |  |  |  |  |  |  |  |  |  |
| **E49** | 121.6 | 179.3 | 59.3 | 29.4 | 36.3 |  | 183.7 |  |  |  |  |  |  |  |  |  |  |
| **Y50** | 119.9 | 177.1 | 62.6 | 38.6 |  |  |  | 132.7 |  |  |  | 157.8 |  |  |  |  |  |
| **Q51** | 117.4 | 180.8 | 60.2 | 27.3 | 33.0 |  | 179.2 |  |  |  |  |  |  |  | 112.9 |  |  |
| **S52** | 116.0 | 175.7 | 63.5 | 62.0 |  |  |  |  |  |  |  |  |  |  |  |  |  |
| **K53** | 122.2 | 178.9 | 58.2 | 32.0 | 24.9 |  | 27.4 |  | 42.7 |  |  |  |  |  |  |  | 32.1 |
| **L54** | 120.8 | 179.5 | 57.9 | 41.0 | 26.4 |  | 25.1 | 21.5 |  |  |  |  |  |  |  |  |  |
| **S55** | 113.2 | 176.1 | 63.1 | 63.7 |  |  |  |  |  |  |  |  |  |  |  |  |  |
| **E56** | 122.1 | 177.6 | 61.5 | 31.5 | 39.2 |  | 182.1 |  |  |  |  |  |  |  |  |  |  |
| **Y57** | 119.1 | 176.9 | 62.7 | 39.7 |  |  | 132.0 | 132.5 | 118.3 |  |  |  |  |  |  |  |  |
| **T58** | 111.1 | 176.3 | 66.2 | 68.7 |  | 22.0 |  |  |  |  |  |  |  |  |  |  |  |
| **L59** | 123.7 | 178.5 | 57.9 | 40.3 | 26.3 |  | 28.1 | 22.2 |  |  |  |  |  |  |  |  |  |
| **Y60** | 124.1 | 177.0 | 59.1 | 36.7 | 129.3 |  | 132.0 | 134.1 | 116.3 | 118.3 |  |  |  |  |  |  |  |
| **R61** | 117.5 | 180.5 | 56.8 | 31.1 | 25.5 |  | 42.5 |  |  |  |  | 159.7 |  |  |  | 80.6 |  |
| **N62** | 119.2 | 176.2 | 56.9 | 39.9 | 176.0 |  |  |  |  |  |  |  |  |  | 111.6 |  |  |
| **A63** | 123.9 | 180.2 | 56.0 | 17.6 |  |  |  |  |  |  |  |  |  |  |  |  |  |
| **Q64** | 122.1 | 175.8 | 58.2 | 31.4 | 33.1 |  | 178.3 |  |  |  |  |  |  |  | 122.6 |  |  |
| **S65** | 110.1 | 179.4 | 60.7 | 63.6 |  |  |  |  |  |  |  |  |  |  |  |  |  |
| **N66** | 117.3 | 177.1 | 55.9 | 37.3 | 174.1 |  |  |  |  |  |  |  |  |  | 110.2 |  |  |
| **T67** | 116.9 | 174.7 | 69.1 | 68.6 |  | 21.1 |  |  |  |  |  |  |  |  |  |  |  |
| **V68** | 120.7 | 176.6 | 67.3 | 32.2 | 21.0 | 22.7 |  |  |  |  |  |  |  |  |  |  |  |
| **K69** | 118.8 | 177.1 | 58.5 | 32.7 | 25.5 |  | 28.1 |  | 42.5 |  |  |  |  |  |  |  | 33.6 |
| **V70** | 118.8 | 179.8 | 66.9 | 32.0 | 22.2 | 24.2 |  |  |  |  |  |  |  |  |  |  |  |
| **I71** | 118.0 | 177.7 | 61.9 | 34.5 | 27.1 | 17.8 | 7.9 |  |  |  |  |  |  |  |  |  |  |
| **K72** | 123.5 | 178.4 | 61.3 | 30.8 | 24.9 |  | 29.5 |  | 42.0 |  |  |  |  |  |  |  | 32.3 |
| **D73** | 119.1 | 180.8 | 57.5 | 39.4 | 179.3 |  |  |  |  |  |  |  |  |  |  |  |  |
| **V74** | 124.9 | 176.9 | 66.3 | 31.9 | 21.5 | 25.1 |  |  |  |  |  |  |  |  |  |  |  |
| **D75** | 119.6 | 177.3 | 55.3 | 38.1 | 172.2 |  |  |  |  |  |  |  |  |  |  |  |  |
| **A76** | 121.4 | 179.9 | 54.8 | 18.3 |  |  |  |  |  |  |  |  |  |  |  |  |  |
| **A77** | 121.4 | 180.1 | 54.7 | 18.2 |  |  |  |  |  |  |  |  |  |  |  |  |  |
| **I78** | 119.8 | 178.3 | 65.1 | 38.3 | 28.3 | 18.4 | 15.2 |  |  |  |  |  |  |  |  |  |  |
| **I79** | 117.0 | 180.0 | 63.5 | 38.1 | 31.5 | 17.2 | 15.2 |  |  |  |  |  |  |  |  |  |  |
| **Q80** | 119.7 | 177.2 | 57.9 | 28.3 | 33.6 |  | 180.7 |  |  |  |  |  |  |  |  |  |  |
| **N81** | 115.4 | 175.4 | 52.7 | 37.9 | 176.0 |  |  |  |  |  |  |  |  |  | 111.4 |  |  |
| **F82** | 118.3 | 175.2 | 56.5 | 38.2 | 140.3 |  | 129.6 |  |  |  |  |  |  |  |  |  |  |
| **R83** | 117.6 | 181.5 | 57.3 | 31.9 | 27.6 |  | 43.7 |  |  |  |  |  |  |  |  | 84.3 |  |

Table S3. Source of primary sequences for multiple sequence alignment.

|  | **Protein name** |  | **Bacterial organism** |  | **UniProt identifier** |  | **Length** |  |
| --- | --- | --- | --- | --- | --- | --- | --- | --- |
|  |  |  |  |  |  |  |  |  |
| **Cluster I** | | | | | | | | |
|  | MxiH |  | *Shigella flexneri* serotype 6 |  | Q6XVY0_SHIFL |  | 83 |  |
|  | MxiH |  | *Shigella flexneri* serotype 2 |  | MXIH_SHIFL |  | 83 |  |
|  | cip-c |  | *Chromobacterium violaceum* |  | Q7NVC1_CHRVO |  | 84 |  |
|  | PrgI |  | *Salmonella typhimurium* |  | PRGI_SALTY |  | 80 |  |
|  | BsaL |  | *Burkholderia pseudomallei* |  | Q63K18_BURPS |  | 89 |  |
|  | EprI |  | *Escherichia coli* |  | Q8X2T4_ECO57 |  | 79 |  |
|  | YsaG |  | *Sodalis glossinidius* |  | Q6R8E1_SODGL |  | 85 |  |
|  |  |  |  |  |  |  |  |  |
| **Cluster II** | | | | | | | | |
|  | AscF |  | *Aeromonas hydrophila* AH1 |  | Q1EHA3_AERHY |  | 87 |  |
|  | AscF |  | *Aeromonas veronii* |  | Q6WG33_9GAMM |  | 85 |  |
|  | AscF |  | *Aeromonas hydrophila* AH3 |  | Q699P8_AERHY |  | 81 |  |
|  | PscF |  | *Pseudomonas aeruginosa* |  | PSCF_PSEAE |  | 85 |  |
|  | YscF |  | *Yersinia pestis* |  | O68691_YERPE |  | 87 |  |
|  | YscF |  | *Yersinia enterocolitica* |  | YSCF_YEREN |  | 87 |  |
|  | SctF |  | *Photorhabdus luminescens* |  | Q7N0U8_PHOLL |  | 84 |  |
|  | YscF |  | *Vibrio parahaemolyticus* |  | Q87P24_VIBPA |  | 82 |  |
|  |  |  |  |  |  |  |  |  |
| **Other sequences** | | | | | | | | |
|  | EscF |  | *Escherichia coli* |  | Q7DB83_ECO57 |  | 73 |  |
|  | EscF |  | *Citrobacter rodentium* |  | Q93FJ3_CITRO |  | 73 |  |
